# Selection on a Subunit of the NURF Chromatin Remodeler Modifies Life History Traits in a Domesticated Strain of *Caenorhabditis elegans*

**DOI:** 10.1371/journal.pgen.1006219

**Published:** 2016-07-28

**Authors:** Edward E. Large, Wen Xu, Yuehui Zhao, Shannon C. Brady, Lijiang Long, Rebecca A. Butcher, Erik C. Andersen, Patrick T. McGrath

**Affiliations:** 1 School of Biology, Georgia Institute of Technology, Atlanta, Georgia, United States of America; 2 Department of Molecular Biosciences, Northwestern University, Evanston, Illinois, United States of America; 3 Department of Chemistry, University of Florida, Gainesville, Florida, United States of America; Stanford University Medical Center, UNITED STATES

## Abstract

Evolutionary life history theory seeks to explain how reproductive and survival traits are shaped by selection through allocations of an individual’s resources to competing life functions. Although life-history traits evolve rapidly, little is known about the genetic and cellular mechanisms that control and couple these tradeoffs. Here, we find that two laboratory-adapted strains of *C*. *elegans* descended from a single common ancestor that lived in the 1950s have differences in a number of life-history traits, including reproductive timing, lifespan, dauer formation, growth rate, and offspring number. We identified a quantitative trait locus (QTL) of large effect that controls 24%–75% of the total trait variance in reproductive timing at various timepoints. Using CRISPR/Cas9-induced genome editing, we show this QTL is due in part to a 60 bp deletion in the 3’ end of the *nurf-1* gene, which is orthologous to the human gene encoding the BPTF component of the NURF chromatin remodeling complex. Besides reproduction, *nurf-1* also regulates growth rate, lifespan, and dauer formation. The fitness consequences of this deletion are environment specific—it increases fitness in the growth conditions where it was fixed but decreases fitness in alternative laboratory growth conditions. We propose that chromatin remodeling, acting through *nurf-1*, is a pleiotropic regulator of life history trade-offs underlying the evolution of multiple traits across different species.

## Author Summary

Sex and death are two fundamental concerns of each organism. These traits evolve rapidly in natural populations as animals seek to maximize their fitness in a given environment. For example, in mammals, lifespan, size, and fecundity vary over two order of magnitude. A key observation of evolutionary life history theory is the recognition that there are limited amount of resources available, which creates tradeoffs between competing life functions. By studying a domesticated strain of *C*. *elegans*, we identify a beneficial mutation that regulates a number of life history tradeoffs. This mutation affects a subunit of the NURF chromatin remodeling complex. Our work suggests that NURF is a master regulator of life history tradeoffs through epigenetic regulation, and a target of evolution.

## Introduction

Organisms are faced with limited resources to invest in their growth, survival, and offspring. Because they cannot dedicate unlimited energy to all traits, they must prioritize energy distribution depending on their environment and sexual partners [1]. Trade-offs represent the combined change in fitness when a beneficial change in one trait is linked to a detrimental trait in another. For example, the benefit of increasing the number of offspring that are produced must be balanced by the cost paid in survival of the parent organism. Life-history theory seeks to understand how an organism’s life-history traits–including reproductive timing and behavior, lifespan, growth rate, and post-reproductive behavior–have been shaped by sexual and natural selection using key concepts such as trait value, trait costs, environmental predictability, and environmental stability [2, 3]. Trade-offs are not absolute, but depend on an animal’s given environment [4], indicating these traits should change as animals speciate and explore new niches. Empirical evidence confirms that life-history traits evolve very rapidly.

**Fig 1:**
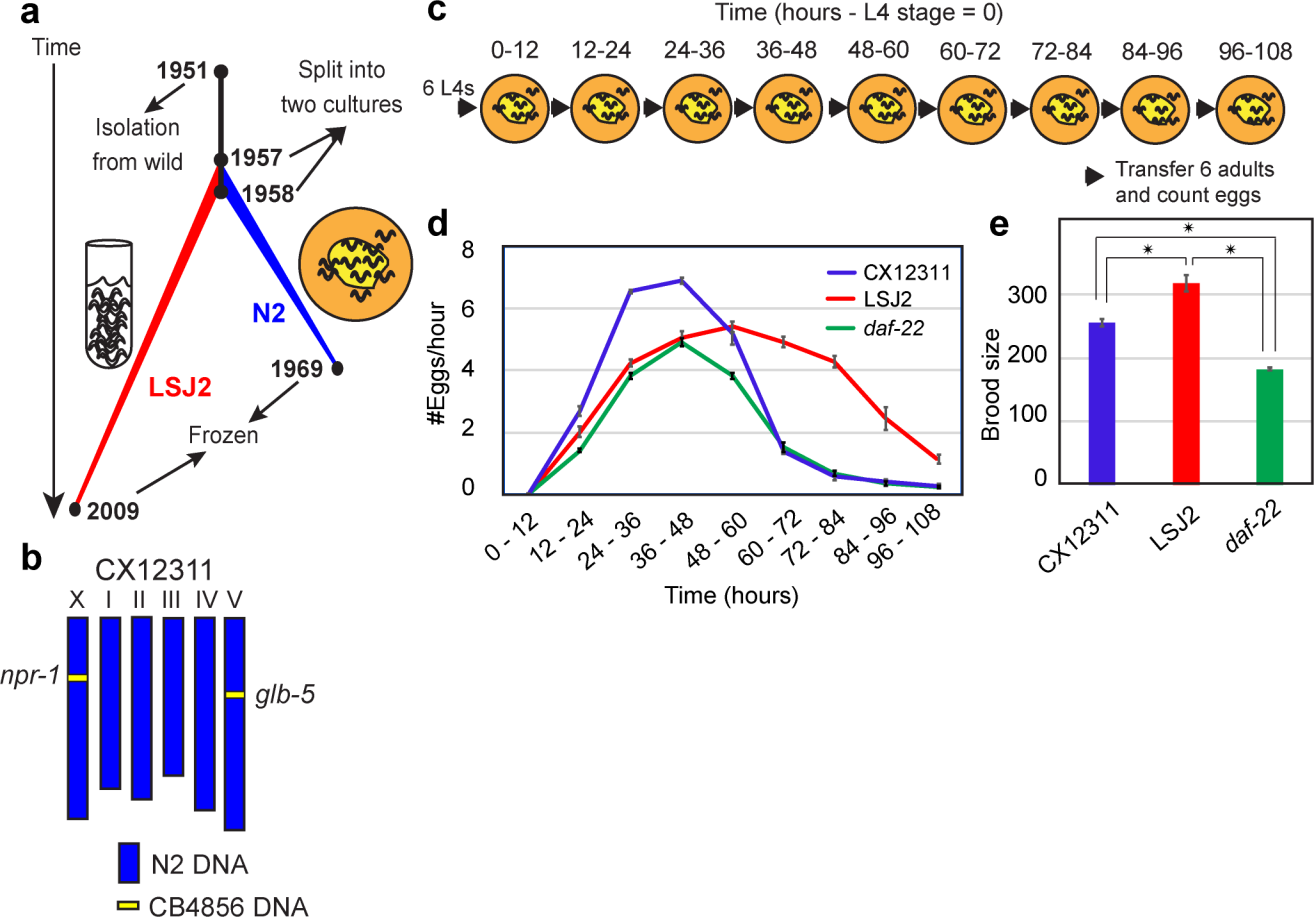
Laboratory adaptation of *C*. *elegans* strains has resulted in modified reproductive rate and timing. **a.** History of the *C*. *elegans* strains N2 and LSJ2 following isolation from the wild (Bristol, England). LSJ2 was grown in liquid axenic culture whereas N2 was propagated on agar plates. **b.** Schematic of CX12311 genetic background containing ancestral alleles of *npr-1* and *glb-5* backcrossed from CB4856. CB4856 is a wild strain isolated from Hawaii. **c.** Schematic of egg-laying experiments performed in **d** and **e**. **d.** Averaged egg-laying rate of the CX12311, LSJ2, and *daf-22* strains starting from the L4 stage. *daf-22* encodes an enzyme necessary for synthesis of ascaroside pheromones. **e.** Total number of eggs laid per animal for the three strains from **d**. Error bars in **d** and **e** represent s.e.m.

Most life-history theories focus on phenotypic traits. However, given that these tradeoffs are ultimately genetically determined, it is important to understand their genetic underpinnings to understand how life-history traits are co-regulated and also how species-level differences in these traits emerge. As with most biological traits, however, few causative alleles have been found that influence life-history traits. As a more tractable model to understand how genetic variants impact a trait, we are focusing our studies on two *C*. *elegans* strains, N2 and LSJ2, derived from the same hermaphrodite isolated in 1951 [5]. Although initially genetically identical, the two strains were separated into distinct cultures of either solid or liquid media in Ellsworth Dougherty’s laboratory in Richmond, California sometime between 1957 and 1958 (**Fig 1A**) [5]. N2 was cultured for approximately 15 years on agar plates seeded with *E*. *coli* bacteria. Bacteria are *C*. *elegans’* natural food source [6], so these growth conditions represent a rich environment for the animals. LSJ2 was cultured for approximately 50 years in liquid, axenic culture consisting of soy-peptone extract supplemented with beef liver extract [7]. This food source is very unnatural for *C*. *elegans*, so these growth conditions likely represent a poor nutrient environment for the animals. Both strains were eventually cryopreserved and the two genomes sequenced. We found that 94 new mutations were fixed in the N2 lineage and 188 new mutations were fixed in the LSJ2 lineage [8]. This genetic diversity is almost three orders of magnitude lower than the genetic diversity between wild strains of *C*. *elegans* [9–11], and four orders of magnitude lower than the genetic diversity between two humans [12], making identification of causative mutations through QTL mapping feasible. Despite this low level of genetic diversity, a large number of phenotypic differences distinguish the two strains. A total of four quantitative trait nucleotides (QTNs) have been identified in these strains to date, providing empirical evidence linking variation in a neuropeptide receptor activity to multimodal changes in social behavior [13, 14], variation in sensory gene deployment with specific chemosensory responses [5, 8, 15], and identifying a source of cryptic genetic variation that affects organ development [16].

Here, we study the genetic basis of reproductive differences between the N2 and LSJ2 strains at five different timepoints. We first identify age-dependent differences in reproductive rate between these two strains. This difference is caused in part by a small deletion fixed in the LSJ2 lineage in the 3’ end of the *nurf-1* gene, which encodes the ortholog to the BPTF subunit of the NURF chromatin-remodeling complex. Besides controlling reproductive timing, *nurf-1* also influences additional life-history traits, including growth rate, lifespan, and dauer formation. The mutation is advantageous in the LSJ2 growth conditions, but disadvantageous in the N2 growth conditions. Finally, we map sensitivity to two unrelated anthelmintic drugs and two heavy metals to *nurf-1*, suggesting that *nurf-1* mutants prioritize individual survival over reproduction. Our results suggest that *nurf-1* is a pleiotropic regulator of life-history traits and a target of evolutionary selection.

## Results

### LSJ2 and N2 differ in their rate of egg-laying and total number of offspring

The timing of reproduction represents one of the most prominent life-history trade-offs [17]. In the course of routinely culturing the N2 and LSJ2 strains, we noticed a difference in egg density on bacterial plates, which we assayed quantitatively. The N2 reference strain has acquired previously described genetic variants in the *npr-1* and *glb-5* genes that affect a number of physiological traits [5, 13, 15, 18]. To avoid studying these previously described laboratory adaptations, we utilized a strain, CX12311, which contains ancestral alleles of these two genes backcrossed to an N2 background (**Fig 1B**). To quantify the difference in egg-laying rates between CX12311 and LSJ2, we measured egg-laying rates from hermaphrodites grown on agar plates seeded with *E*. *coli* bacteria starting from the fourth larval stage (L4) and then twice a day for five days (**Fig 1C**). Beginning the experiment with L4 animals allows us to bypass differences in growth rate and focus on the rate of sexual maturity and reproductive timing. These conditions resemble the standard laboratory conditions for culturing the N2 strain. The egg-laying rate for CX12311 was extremely low for the first time-point, reflecting the switch from spermatogenesis to oogenesis [19]. This rate increased for the next three time points, peaking at approximately seven eggs per animal per hour at the 36–48 hour time point and then decreasing until the last time point, when the majority of animals ceased reproduction (**Fig 1D**). The initial increase likely reflects the accumulation of oocytes while the cessation reflects animals running out of sperm for self-fertilization [19]. For the initial four time points, LSJ2 laid fewer eggs than CX12311 animals (**Fig 1D**). However, their rate continued to increase, resulting in a higher egg-laying rate starting from the 48–60 hour time point. These experiments indicate that new mutation(s) affecting reproductive timing have become fixed in the N2 and/or LSJ2 lineage.

We also measured the total fecundity of each strain over the course of their reproductive life. Each LSJ2 hermaphrodite laid ~310 fertilized eggs, whereas the CX12311 hermaphrodites laid ~250 fertilized eggs (**Fig 1D**). The total self-reproductive capacity of a *C*. *elegans* hermaphrodite is limited by the number of self-sperm that are created during the L4 stage, suggesting that these strains are born with a different number of self-sperm, however, we did not directly count self-sperm to confirm this.

We have previously found that pheromones released by *C*. *elegans* act as an important selective pressure in animals grown in liquid axenic media, including the LSJ2 strain [8]. We wondered if impaired pheromone processing in LSJ2 could be involved in the difference in reproductive timing between CX12311 and LSJ2 as pheromones have been reported to impact brood size in certain environmental contexts. To test this hypothesis, we generated two strains containing deletions in the *daf-22* gene in the CX12311 background using the CRISPR-Cas9 system. The DAF-22 protein is required for synthesizing the majority of pheromones released by *C*. *elegans* [20, 21]. For the first four timepoints, *daf-22* animals laid eggs at a rate almost indistinguishable from LSJ2. For the next five timepoints, *daf-22* animals laid eggs at a rate much lower than LSJ2 (**Fig 1D**). As a result their total fecundity was much lower than either LSJ2 or CX12311 (**Fig 1E**). These results are consistent with pheromones failing to stimulate egg-laying in LSJ2 for the first four timepoints. However, LSJ2 and *daf-22* have different temporal patterns of egg laying, suggesting that *daf-22* also affects total fecundity besides just the initial timing of egg production.

### QTL mapping identifies a major peak on chromosome II

**Fig 2:**
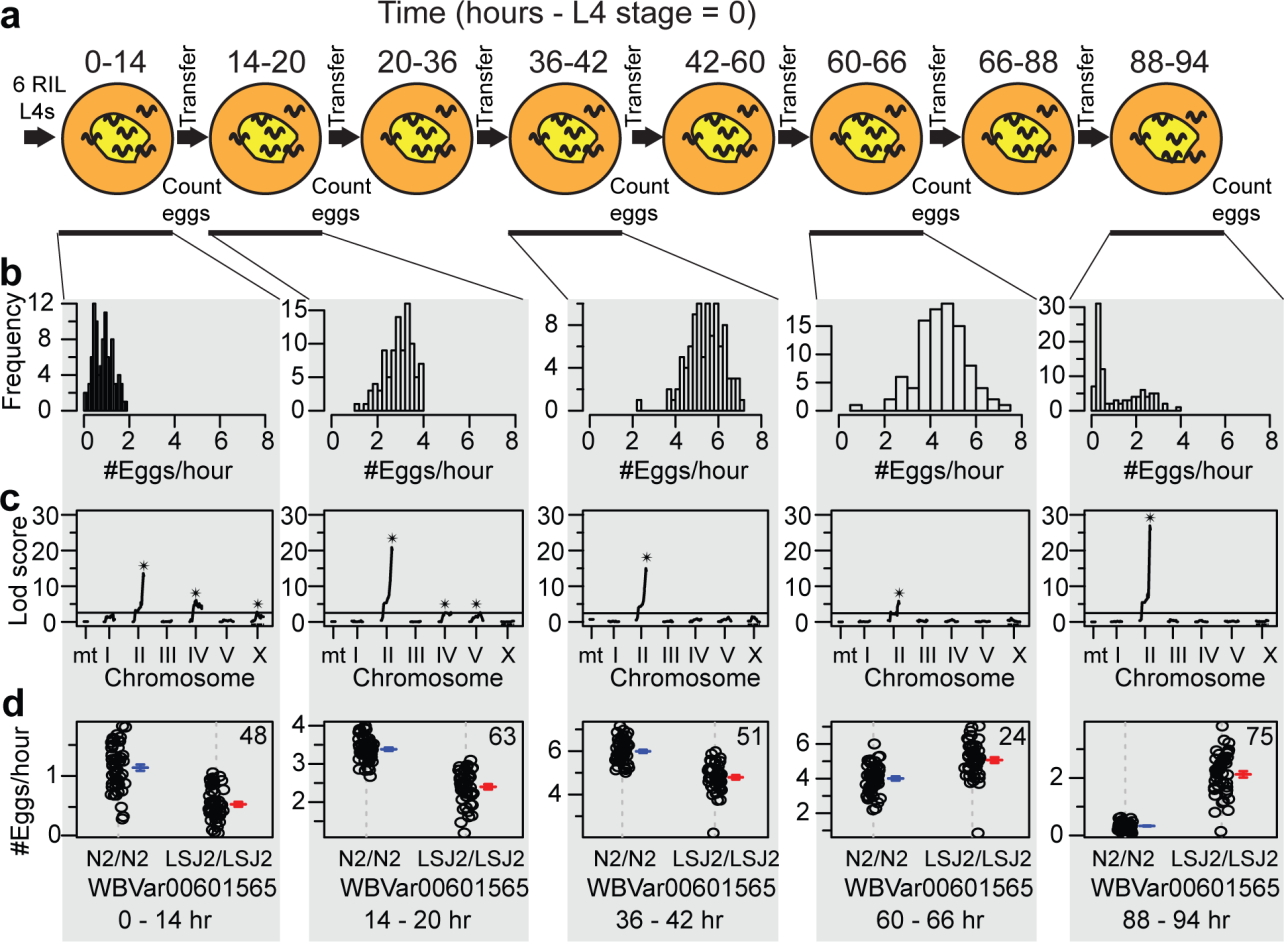
QTL mapping of differences in LSJ2 and CX12311 reproductive rate and timing. **a.** Schematic of egg-laying experiments performed on 94 recombinant inbred lines (RILs) generated between LSJ2 and CX12311. **b.** Histogram of the egg-laying rate measured from 94 RILs at five different time points. **c.** QTL mapping on data from B identified a major effect locus on the right arm of chromosome II at all five time points. Additional smaller effect QTL were identified for a subset of time points on chromosomes IV, V, and X. Stars identify QTL that reach genome-wide significance. **d.** Egg-laying rate of RILs partitioned by their genotype at the genetic variant with the peak LOD score (WBVar00601585) in the major effect QTL on the right arm of chromosome II. The effect of this variant was age dependent. Mean and s.e.m. plotted immediately to the right. Number in the upper right indicates percent of phenotypic variation explained by the QTL.

In order to identify the causative genetic variants responsible for these differences, we performed quantitative trait loci (QTL) mapping using a panel of 94 recombinant inbred lines (RILs) [22]. The egg-laying rates of all RILs were assayed in replicate over the same five time-points (**Fig 2A and 2B**). In total, over 250,000 eggs were counted. QTL mapping on this averaged data identified a highly significant major-effect QTL on the right arm of chromosome II (QTL_II_) that accounted for as little as 24% and as much as 75% of the overall phenotypic variation for a particular day (**Fig 2C and 2D**). Interestingly, the effect of this QTL switched over the course of the animal’s life (**Fig 2D**), changing its direction between day 2 and 3 (*i*.*e*. sign-switching). Therefore, we identified a QTL on chromosome II with variable effect size that both decreases and increases the egg-laying rate over the entire reproductive life history of the animal. Additional QTLs of smaller effect were also identified at various timepoints on chromosomes IV, V, and X.

**Fig 3:**
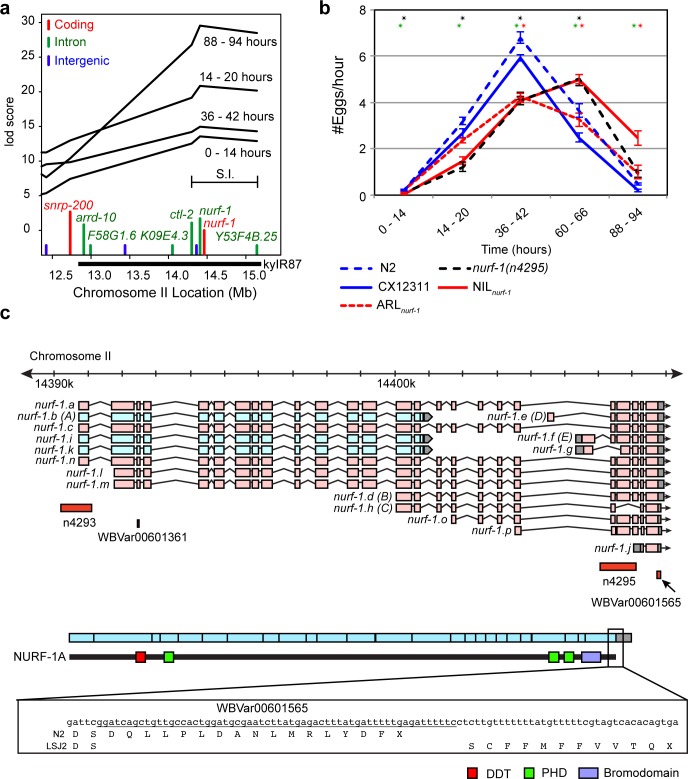
The major-effect QTL on II is partially explained by a 60 bp deletion in *nurf-1*. **a.** QTL mapping at four time points plotted on the right arm of chromosome II. Bayesian significance interval is shown as a bar (S.I.). Genetic variants between LSJ2 and N2 plotted along the x-axis (color indicates their location; height of bar has no significance). The boundaries of an introgressed region created surrounding this QTL are shown below the x-axis (*kyIR87*). **b.** Egg-laying rate of N2, CX12311, NIL_*nurf-1*_, ARL_*nurf-1*_, and *nurf-1(n4295)*. NIL_*nurf-1*_ is an introgression of LSJ2 DNA near *nurf-1* (*kyIR87*, see **a**) into CX12311. ARL_*nurf-1*_ is the result of engineering the LSJ2 deletion in *nurf-1* into the CX12311 background using CRISPR/Cas9. Two independent ARL_*nurf-1*_ strains were constructed and data show the average of both strains. The *nurf-1(n4295)* strain was constructed in the N2 background. Black stars (top of triangle) indicate significant differences between N2 and *nurf-1(n4295)*. Green stars (bottom left) indicate significant differences between CX12311 and NIL_*nurf-1*_. Red stars (bottom right) indicate significant differences between CX12311 and ARL_*nurf-1*_. Error bars represent S.E.M. **c.** Genomic region surrounding *nurf-1* indicating the location of two canonical mutagenesis-derived alleles (*n4293*, *n4295*), N2-fixed SNP (WBVar00601361), and LSJ2-fixed 60 bp deletion (WBVar00601565), predicted mRNA and protein product, and predicted effect on protein sequence of the LSJ2 deletion. Isoforms that are predicted to be affected by the WBVar00601565 deletion are colored in light red.

The QTL_II_ was mapped to a ~1 Mb region containing five genetic differences–single nucleotide variants (SNVs) in the introns of *ctl-2*, *Y53F4B*.*26*, and *nurf-1*, an SNV mutation in an intergenic region, and a 60 bp deletion in the 3’ coding region of *nurf-1* (**Fig 3A**). We verified this QTL by introgressing LSJ2 DNA surrounding QTL_*nurf-1*_ into CX12311 (**Fig 3A**). The resultant near-isogenic line (NIL_*nurf-1*_) recapitulated the result of QTL mapping (**Fig 3B**). We focused on the *nurf-1* coding region deletion as a likely candidate for the causative variant. *nurf-1* is an uncommonly complex locus encoding at least 16 isoforms that are orthologs of human BPTF. BPTF is a subunit of the NURF chromatin-remodeling complex, which recognizes multiple histone modifications on nucleosomes and recruits ISWI to remodel nearby nucleosomes (**Fig 3C**) [23, 24]. In Drosophila, a long-form (analogous to the a, c, l, m, and n isoforms) and a 5’ short form (analogous to the b, I, and k isoforms) are known to exist [25]. However, in *C*. *elegans* and *C*. *briggsae*, additional isoforms covering the 3’ end of the *nurf-1* gene have also been identified (d, e, f, g, h, j, o, and p) [23, 26]. Little is known why so many isoforms are necessary for *nurf-1* function and it is still controversial whether the long form even exists in nematodes [23, 26]. The 60 bp deletion in the LSJ2 strain affects 13 out of 16 *nurf-1* isoforms, removing the 3’ coding region of *nurf-1*, the stop codon, and 8 bp of the 3’ UTR. These changes are predicted to replace the last 16 amino acids of the NURF-1 protein with 11 novel residues (**Fig 3C**). Because the specific *nurf-1* isoforms that are mutated in LSJ2 have not been shown to affect *C*. *elegans* reproduction, we first tested a 1078 bp deletion (*n4295*) in the 3’ end of *nurf-1* (**Fig 3C**) [23]. This strain was created in an N2 background, so we compared its egg-laying rate to N2. This strain was nearly indistinguishable from the NIL_*nurf-1*_ (**Fig 3B**), indicating one or more of these eight isoforms regulate egg laying. We next asked whether the specific LSJ2 60 bp deletion was causal by using CRISPR/Cas9 combined with template-based repair to generate two independent allele replacement lines (ARL_*nurf-1*_) carrying the LSJ2 allele of *nurf-1* in the CX12311 background [27, 28]. The egg-laying rates of these two ARL_*nurf-1*_ strains were significantly different from CX12311 on the third, fourth, and fifth time-points in a direction consistent with the QTL mapping and the NIL strain (**Fig 3B**). However, these strains were also distinguishable from the NIL_*nurf-1*_ strain, showing no significant difference with CX12311 at the first two time points and a smaller effect size than the NIL_*nurf-1*_ during the last two time points. These results suggest that the 60 bp deletion is a causative genetic variant for egg-laying differences between N2 and LSJ2, but additional causative genetic variants exist within the introgressed region (**Fig 3A**). These causative genetic variants could potentially act through the *nurf-1* gene (such as the WBVar00601361 variant found in the *nurf-1* intron), but additional work will be necessary to clarify their identity and role.

### *nurf-1* regulates additional life-history traits

**Fig 4:**
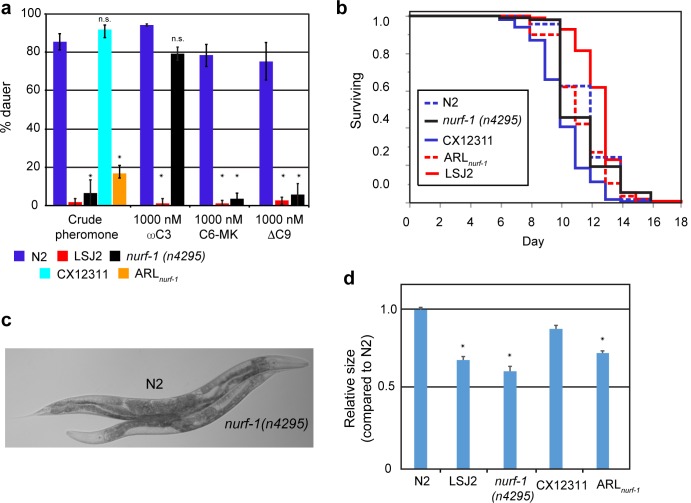
*nurf-1* regulates additional life-history traits. **a.** Dauer formation of the N2, LSJ2, CX12311, ARL_*nurf-1*_, and *nurf-1(n4295)*, strains in response to crude pheromone or N2, LSJ2, and *nurf-1(n4295)* in response to synthesized pheromone components. y-axis indicates the percentage of animals that enter dauer in response to the pheromone concentrations listed on the x-axis averaged from at least fiver replicates. Stars indicate significant differences (p<0.05) from the N2 strain. **b.** Lifespan analysis of the N2, LSJ2, CX12311, ARL_*nurf-1*_ and *nurf-1(4295)* animals. At least three independent replicates containing ~60 worms were used for this analysis. The LSJ2 and ARL_*nurf-1*_ strain were both significantly different (p < 0.05) than CX12311. The *nurf-1(4295)* strain was not significantly different from the N2 strain. **c.** Micrograph comparing the size of N2 and *nurf-1(n4295)* animals. Embryos were isolated from each strain and allowed to hatch and grow on agar plates for 72 hours. **d.** Growth rate of N2, LSJ2, CX12311, ARL_*nurf-1*_ and *nurf-1(n4295)* strains. y-axis shows the average area of each animal measured by videotracking normalized to the N2 strain. Three independent growth plates were analyzed each day and three replicates were performed on different days. Their size was measured 72–75 hours after isolation. Stars indicate significant differences (p<0.05).

We had previously found that the LSJ2 strain was defective for pheromone-induced entry to a diapause state called dauer [8]. Dauer is another example of a life-history trade-off in *C*. *elegans* [29]. Although dauer animals are resistant to a number of stressors and live 5–6 times longer than normal animals, this trade-off comes at the expense of reproduction. We tested the ability of *nurf-1(n4295)* and the *ARL*_*nurf-1*_ animals to enter dauer in response to crude pheromone extracts. *n4295* is a previously generated deletion allele in the 3’ end of *nurf-1* (**Fig 3C**). Although N2 and CX12311 animals readily entered dauer, LSJ2 and *nurf-1* mutant animals did not (**Fig 4A**). *C*. *elegans* release a number of dauer pheromones, which are sensed and transduced by different genetic and neuronal pathways. We tested N2, LSJ2, and *nurf-1 (n4295)* animals to three synthesized components of the dauer pheromone cocktail, ωC3 (ascr#5), C6-MK (ascr#2), and ΔC9 (ascr#3) [30, 31]. *nurf-1* animals entered dauer in response to ωC3 but were reduced in their propensity to enter dauer in response to C6-MK and ΔC9 (**Fig 4A**). This result indicates that the role of *nurf-1* in dauer formation is selective for individual pheromones, suggesting a role in the neural circuit that senses and transduces the C6-MK and ΔC9 pheromones [32].

We next tested the role of *nurf-1* in lifespan by examining the N2, CX12311, LSJ2, *nurf-1(n4295)*, and ARL_*nurf-1*_ strains at 25°C. In these conditions, the average lifespan of CX12311 animals is approximately 12.2 days, and the lifespan of LSJ2 animals was extended by a few days (14.6 days) (**Fig 4B**). The ARL_*nurf-1*_ strain fell in between the two parental strains (13.3 days), indicating that *nurf-1* has an effect on lifespan, but additional genetic variants between LSJ2 and N2 also contribute to lifespan differences. We also tested the N2 and *nurf-1* mutant strain but found no significant difference between their lifespan. This suggests that the ancestral *npr-1* allele is required for *nurf-1’s* role in regulating lifespan.

Finally, we tested the growth rate of animals by synchronizing animals at hatching followed by video recordings of the animals at 72 hours. These videos were analyzed by custom software to identify the pixel area of the animals normalized to the N2 strain [33] (**Fig 4C and 4D**). Animals increase their volume 100-fold over the course of these experiments. This analysis revealed that both LSJ2 and *nurf-1* animals grew at a slower rate than N2 or CX12311.

### The *nurf-1* deletion is disadvantageous on agar plates but is advantageous in liquid media

**Fig 5:**
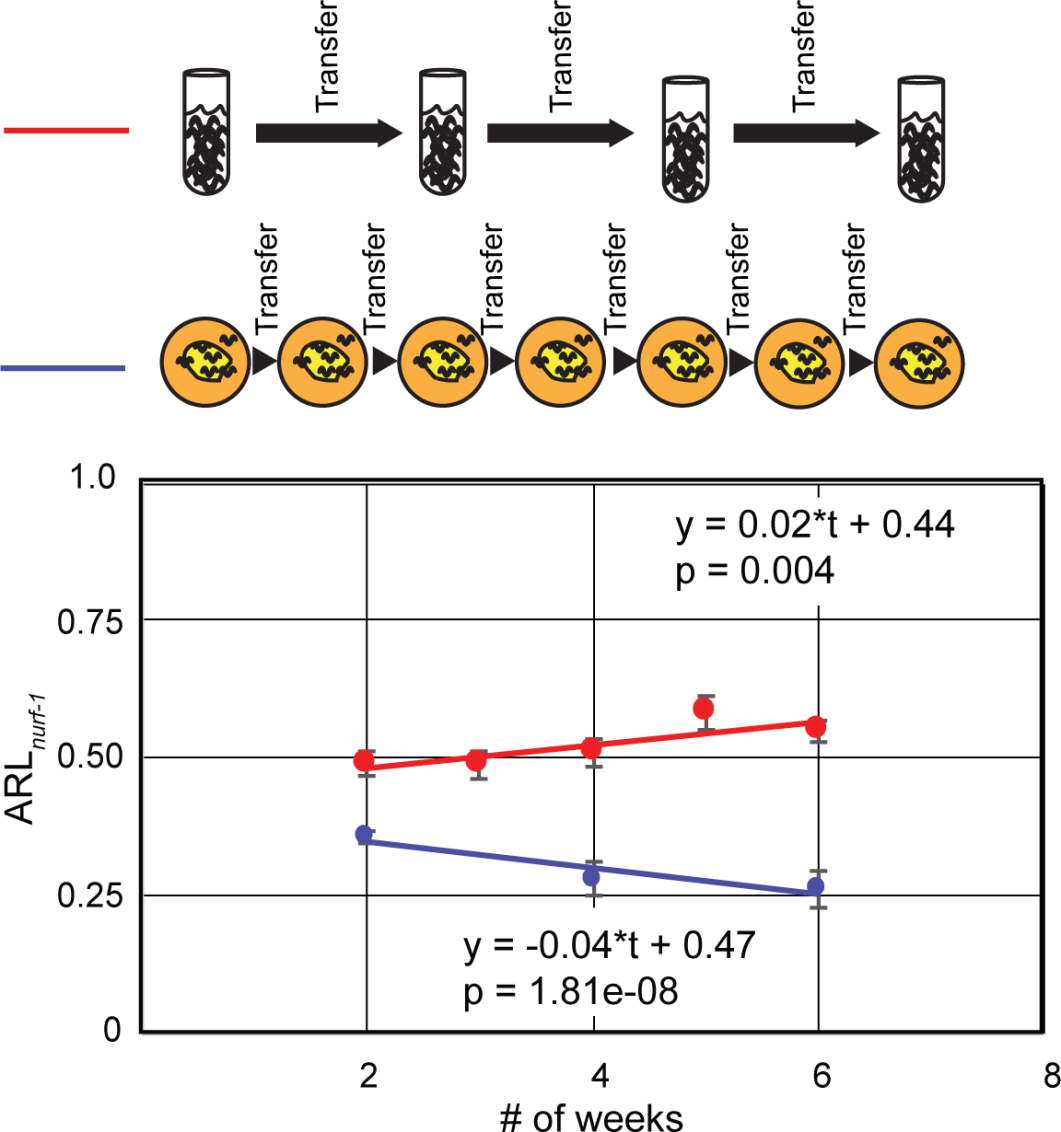
The 60 bp deletion in *nurf-1* is advantageous in liquid axenic media but disadvantageous on agar plates. A diagram of the two competition conditions is shown above the graph. The ARL_*nurf-1*_ strain (PTM88) was competed against CX12311. The fraction of ARL_*nurf-1*_ animals in the population is shown on the y-axis. The equation for a linear regression fit is also shown on the graph. p value indicates significance of the temporal term by ANOVA. Error bars represent standard error.

From an evolutionary perspective, it is unexpected that an allele that reduces early reproductive rate could spread to fixation. While deleterious alleles can spread in populations with small bottlenecks [34], many of the causative genetic variants that have become fixed in the LSJ2/N2 lineages have been shown to have a positive effect on fitness [14, 16]. We tested the effect of the 60 bp deletion directly using competition experiments. First, we competed the ARL_*nurf-1*_ strain against CX12311 animals on agar plates (*i*.*e*. the historical N2 growth conditions) using nine experimental replicates consisting of five competition plates each. Competition plates were seeded with seven animals from each genotype. Once a week for six weeks, after food had been exhausted for approximately three days, 100 to 200 starved L1s were transferred to a new freshly seeded plate. Every two generations, DNA was isolated from the remaining animals following transfer. DNA from the five competition plates for each replicate were pooled together and genotype frequencies were determined using digital PCR. Fitness was then estimated by fitting linear curve to the data points and using ANOVA to determine its significance. These experiments indicated a clear selective advantage for the CX12311 strain (s = 0.08—**Fig 5**). By the end of the six-week experiment, only 25% of the animals carried the 60 bp deletion in *nurf-1*. These experiments indicate that the *nurf-1-*influenced changes in life history are detrimental in standard laboratory growth conditions.

Using a wild strain of *C*. *elegans* as an outgroup, we previously determined that the 60 bp deletion was fixed in the LSJ2 lineage We reasoned that the 60 bp mutation could have been advantageous in the axenic soy peptone- beef liver growth medium in which LSJ2 arose. To test this hypothesis, ARL_*nurf-1*_ animals were competed against CX12311 animals in axenic media. One hundred animals of each strain seeded into six initial cultures. Every two weeks, 1000 to 2000 animals were transferred to fresh media and DNA was extracted from the remaining animals. In these conditions, the 60 bp deletion resulted in a significant advantage for the animals (s = 0.04—**Fig 5**). These experiments suggest that the life-history changes induced by *nurf-1* are advantageous in the environment in which they originated.

### Differences in sensitivities to four abiotic perturbations also map to *nurf-1*

**Fig 6:**
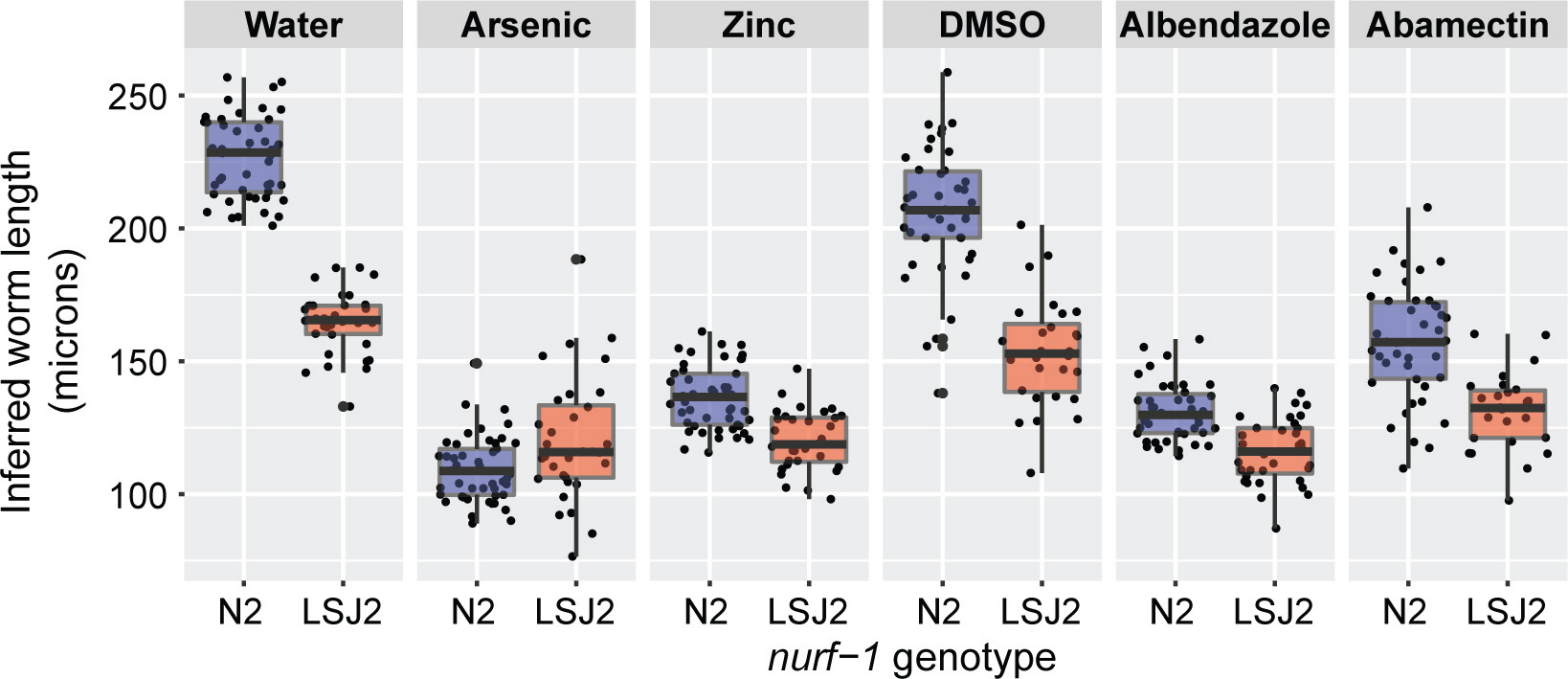
Average length of a population of RILs partitioned by their genotype at *nurf-1* (WBVar00601585) as measured by a COPAS BIOSORT in response to a variety of abiotic stressors. Time of flight is correlated with average length of the animals. Water and DMSO are controls for solvents necessary to dissolve the various abiotics.

Differential stress responses are frequently observed in strains with divergent fitness trade-offs [35]. A strain that commits resources to generate more robust but fewer offspring will typically have higher stress tolerance. By contrast, a strain that commits resources to generate a large number of offspring will have lower stress tolerance. We measured fecundity and population growth rate of the same panel of 94 RILs constructed between CX12311 and LSJ2 parents after exposure to anthelmintic compounds and heavy metals to assess whether these strains differed in their abiotic stress responses. In control conditions (water or DMSO), we found a strong effect of *nurf-1* on mean animal size (**Fig 6**). However, the effect of *nurf-1* was almost completely abrogated in the presence of two anthelmintics (albendazole and abamectin) and two heavy metals (arsenic and zinc) (**Fig 6**). This strong gene-by-stress interaction (**S1 Fig**) suggests that variation in *nurf-1* has generic effects in the presence of abiotic compounds. The N2 allele of *nurf-1* promotes faster growth, but is less able to cope with environmental stressors. Unlike growth rate, the effect of *nurf-1* on number of eggs laid was independent of abiotic perturbations (**S1 Fig**). This suggests that the *nurf-1* animals continue to deprioritize reproductive rate irrespective of their environment.

## Discussion

Our results indicate that a discrete shift in environment resulted in the evolution of multiple traits in LSJ2 animals, including reproductive timing, dauer formation, lifespan, survival, and growth rate. These traits are all classical life history trade-off traits that are indicators for how an organism distributes its resources. LSJ2 animals prioritize individual survival over reproductive speed, as they live longer, grow slower, and are less affected by various drugs and stressors at the cost of laying eggs later on in life. *A priori*, we did not expect LSJ2 to undergo life history changes. Retrospectively, however, these changes may be linked to the unnatural and poor nutritional value of liquid axenic media, where animals reproduce at an order of magnitude lower rate [7]. A strain of the related species *C*. *briggsae* that grew in the same media evolved similar changes to its life history traits, including growth rate and reproductive timing changes [36]. These observations are all consistent with the hypothesis that growth in this media creates selective pressure on life history traits.

Although an extensive literature on life history differences among species exists, little is known about the genetic causes of these changes. We identified a beneficial 60 bp deletion in *nurf-1*, which encodes an ortholog of human BPTF, as a large-effect, pleiotropic regulator of many of the LSJ2 life-history changes. BPTF is a subunit of NURF, a chromatin-remodeling complex that modifies transcription and promotes proliferation and differentiation of a number of tissues in an organism-specific manner [37]. To our knowledge, BPTF/NURF has not previously been described as a regulator of life-history traits. Mechanistically, there are a number of ways it could accomplish this regulation. For example, NURF-1/BPTF could control energy distributions–shunting energy towards survival naturally changes the remaining life history traits. However, in other organisms, these life history axes can vary independently, suggesting energy constraints aren’t sufficient to completely explain their coupling. Alternatively, the life history traits could be linked together in logical ways through concordant NURF-1/BPTF regulation of the different tissues/cells that control these traits. In this scenario, NURF-1 would act as a master regulator of life-history tradeoffs. This latter role is mainly speculative, however, and will require molecular and cellular characterization of *nurf-1* function to determine how its regulation occurs.

Orthologs of NURF-1 have been reported to regulate a large number of disparate traits, however, little is known about the underlying logic that link these traits together. Regulation of life history tradeoffs could be a useful lens to consider NURF-1/BPTF function in other species if its role as defined in *C*. *elegans* has been conserved throughout evolution. It is intriguing that many traits controlled by NURF-1 orthologs in other species could also be interpreted as life-history tradeoffs. For example, both *C*. *elegans* and the related nematode *Caenorhabditis briggsae* have independently evolved self-fertilizing hermaphroditism, which requires their gonads to produce male gametes before switching to female gamete production. Major alterations in the use of NURF-1 in the *C*. *briggsae* germline result in a dependence on *nurf-1* for the sperm/oocyte decision [26]. This evolutionarily recent gain of function for *nurf-1* can be seen as an effect on the timing of the conversion from male to female gametes as a life-history trait–it determines the total number of progeny that can be produced through self-fertilization at a cost of when reproduction can begin [38]. In *Drosophila melanogaster*, *Nurf301* (the ortholog of *nurf-1*) regulates heat shock response, hemocyte proliferation/development, spermatogenesis, oogenesis, and metamorphosis [25, 39–41]. These traits are all life history traits–heat shock response and hemocyte production (*i*.*e*. invertebrate immune system cells) are energetic investments into organismal survival; spermatogenesis and oogenesis are energetic investments into reproduction; and metamorphosis profoundly influences mortality rates, resource intake and the ability to reproduce [17]. In mice, *Bptf* regulates thymocyte maturation [42], the precursor to T-cells that form part of the innate and adaptive immune system that regulates individual survival.

One of the unusual features of *nurf-1* is the large number of isoforms it expresses. Changes in alternate splicing could be particularly relevant for the evolution of BPTF/NURF-1 biological function or new biological functions more generally. The orthologous relationship between human BPTF and *C*. *elegans* NURF-1 shows that there has been little evolutionary pressure to diversify its protein function. However, although human BPTF is expressed from a large number of transcripts (22 predicted by GenBank), no one-to-one relationship between NURF-1 and BPTF isoforms exists. This suggests that evolution has diversified NURF-1/BPTF function by creating/removing specific isoforms of the protein. Isoform-specific evolution combines features of both *cis-* and *trans*-regulation. Cellular expression of individual transcripts can be changed by evolution of its promoter region or by modifying the tissues where alternative splicing occurs. Evolution of new transcripts also functionally changes the protein by the addition or loss of protein residues. Why would *nurf-1* need to evolve both? This complexity could result from a combinatorial need of NURF-1 to regulate transcription in a number of different tissue types (requiring *cis* regulatory evolution) while simultaneously gaining or losing interactions with different transcription factors expressed in these tissues (requiring *trans* regulatory evolution).

While we have used the term NURF-1 very loosely for brevity, there are actually a large number of isoforms that are encoded by the *nurf-1* locus. The LSJ2 deletion that was identified here is predicted to regulate 13 of these different isoforms. While most (12 of 13) of these isoforms contain the domains necessary for interacting with nucleosomes, many lack the canonical domain thought to be required for interactions with ATPase component of NURF responsible for remodeling nucleosomes. In fact, it is unclear in *C*. *elegans* whether the longest isoforms of *nurf-1* (a, c, k, and n), necessary for producing the full form of NURF complex, even exist. While work on the 5’ isoforms of *nurf-1* have demonstrated a role for these isoforms in vulval development and fertility, this report identifies biological traits (dauer, lifespan, reproductive rate and timing, and growth rate) that are associated with the 3’ isoforms.

Life history differences are often considered at the level of species. However, most life-history traits are extremely plastic over the course of the animal’s lifetime and can respond to the different environments animals encounter. Life-history plasticity has been observed in snails, for example, in response to the presence of crawfish predators [43]. Pheromones also regulate life-history tradeoffs: density pheromones induce *C*. *elegans* to enter a diapause state called dauer that prioritizes survival over reproduction, alarm pheromones sensed by cane toads modify their size at metamorphosis [44, 45], and sexual pheromones modify a number of male behaviors that result in prioritizing reproduction over individual survival. In *C*. *elegans*, pheromones also regulate reproductive recovery from stress and total lifespan [46, 47]. We also found that *daf-22* mutants, which are unable to synthesize and secrete pheromones, show differences in reproductive timing (**Fig 1D**). Little is known about the signaling pathways connecting pheromones with many of these traits. We suggest that NURF-1 could mediate some or all of these effects of pheromones on life-history plasticity. In *C*. *elegans*, alternative developmental histories triggered by dauer pheromones result in a cellular memory encoded through histone modifications that regulates a number of life-history traits [48]. Interestingly, the particular down-regulated histone modifications, H3K4me3 and H4ac, are consistent with the canonical histone recognition sites of BPTF (H3K4me3 and H4K16ac) [24]. NURF-1/BPTF could act as a connection point between an animal’s development and current life-history traits activing via alternatively marked nucleosomes. In either case, the identification of *nurf-1* represents a genetic handle to understand life history regulation.

## Methods

### Strains

Strains were cultivated on agar plates seeded with *E*. *coli* strain OP50 at 20°C [49].

Strains used in this study are: N2, LSJ2, CX12311 *kyIR1(V*, *CB4856>N2); qgIR1(X*, *CB4856>N2)*, MT13649 *nurf-1 (n4295)*, PTM88 (ARL_*nurf-1*_) *nurf-1(kah3); kyIR1(V*, *CB4856>N2); qgIR1(X*, *CB4856>N2)*, PTM93 (ARL_*nurf-1*_) *nurf-1(kah5); kyIR1(V*, *CB4856>N2); qgIR1(X*, *CB4856>N2)*, PTM66 (NIL_*nurf-1*_) *kyIR87(II*, *LSJ2>N2); kyIR1(V*, *CB4856>N2)*

RIL strains used in this study are sequentially: CX12312–CX12327, CX12346–CX12377, CX12381–CX12388, CX12414–CX12437, and CX12495-CX12510. These strains were generated and described in a previous study [8].

### Egg-laying assays

All egg-laying assays were carried out at 20°C using standard 5.5 cm NGM plates seeded with the OP50 strain of *Escherichia coli*. Two OP50 concentrations were used to generate either transfer or experimental plates. A glycerol stock of OP50 was used to streak an LB plate and a single colony was cultured overnight. The overnight culture was used to inoculate 200 ml of LB for 4–6 hours of growth at 37°C with shaking. Approximately 1 ml of this culture was swirled around on an unseeded plate to generate a uniform lawn or 300 μl of OP50 was used for transfer plates. For experimental plates, the overnight OP50 culture was concentrated via centrifugation to an OD_600_ of 2.0 and this culture was used for seeding experimental plates with 70 μl aliquots. Both transfer and experimental plates were prepared the week of the assay and left at 22.5°C 18–24 hrs following seeding. Plates were then placed at 4°C until the day of the assay and warmed to 20°C for four hours before each time point.

Nematodes were cultured at least three generations prior to the beginning of the assay. Six young adult hermaphrodite nematodes were then placed on multiple transfer plates two days before the assay and six fourth larval stage (L4) nematodes were transferred to the first 70 μl experimental plate. For picking L4 worms, we identified animals with the "Christmas tree stage" vulvas, which corresponds with mid-L4 stages L4.4, L4.5, and L4.6. This time of this transfer to the first experimental plate represents time 0.

For QTL mapping, L4 hermaphrodites from 94 RIL strains were transferred from the transfer plate to experimental plates and then transferred to 70 μl experimental plates between intervals. For the first two assays, three time points (0–14, 14–20, and 36–42 hours) were measured. For the last three assays, five time points were measured. Laid eggs were allowed to develop to the L4 stage and placed at 4°C for counting.

Egg-laying assays for **Fig 3B** were performed essentially the same way as QTL mapping. Ten replicates were assayed for each strain. For **Fig 1D**, animals were transferred directly between experimental plates. Six replicates were assayed for each strain.

### Statistics

Significant differences between means were determined using unpaired, two-tailed t-tests assuming equal variance.

### QTL Mapping

The average of five egg-laying counts was used as the phenotype for five different time points in combination with 192 previously genotyped SNPs [8]. R/qtl was used to perform a one-dimensional scan using marker regression on the 192 markers [22]. The significance threshold (p = 0.05) was determined using 1000 permutations. For **Fig 2D**, the plotPXG function was used to show the effect of the *nurf-1* marker. The effect-size of the *nurf-1* marker was estimated using fitqtl with a single QTL.

### CRISPR-Cas9 strains

We generated the ARL_*nurf-1*_ strain following the published co-conversion CRISPR method to simultaneously edit the *dpy-10* gene (as a visual marker) along with *nurf-1* using single-stranded oligonucleotidess as repair templates [27]. All sgRNAs were cloned into a subclone of pDD163 containing the U6 promoter to drive sgRNAs in the germline [28]. For the *dpy-10* gene, we used the previously published sgRNA and repair oligo. For the *nurf-1* gene, we designed an sgRNA to target the 5’-TTCGGATCAGCTGTTGCCAC(TGG)-3’ protospacer/PAM site found in the LSJ2 60 bp deletion. We used single-stranded oligonucleotide

5’-TCTATCAGAAAGCGTGTCCAGTCGGAAAGCCAGCGAACTGTCGACTCGTTGGATATCGATTCCTC

TTGTTTTTTTATGTTTTTCGTAGTCACACAGTGACTTTTCACTTGTTACGTTGACAATGT -3’

as a repair construct. To drive Cas9 in the germline, we subcloned P_*eft-3*_::Cas9 from pDD162 into a separate vector. We injected 50 ng/ul P_*eft-3*_::Cas9, 25 ng/ul *dpy-10* sgRNA, 500 nM dpy-10(cn64) repair oligo, 25 ng/ul *nurf-1* sgRNA, and 500 nM LSJ2 *nurf-1* repair oligonucleotide into CX12311 animals. We genotyped 113 F1 roller animals by PCR using the primers

5’- ACATTATACGAAGTTATGTCGTCAAACTTTGCATTTG-3’ and

5’-CATCTTCATAATTCCAACGGAAACCAAG-3’

followed by digestion with PvuII (a site which is removed by the 60 bp LSJ2 deletion). We identified a single PvuII resistant band, however, Sanger sequencing showed that this F1 animal contained a 10 bp deletion of 5’-AGCTGTTGCC-3’ replaced by 5’-GA-3’, indicating that this lesion resulted from a NHEJ event that disrupted the PvuII site as opposed to our targeted HR from the *nurf-1* oligo.

We hypothesized that due to the 60 bp deletion, the flanking regions on the single-stranded oligonucleotide were not long enough to initiate homologous repair. We next generated a strain, PTM91, containing an extrachromosomal array of the LSJ2 *nurf-1* 3’ region by injecting a PCR product generated using the 5’-GCAATTTGTGAACGACGTGA-3’ and 5’-CCGGTCTCGACACAATTTTT-3’ primers along with a P_*elt-2*_::GFP co-injection marker into CX12311 animals. We injected the co-conversion injection mix described above into these animals and again singled 80 F1 roller or roller/dumpy animals and genotyped them as above. We identified two PvuII-resistant bands, which Sanger sequencing showed was due to presence of the LSJ2 60 bp deletion in both strains. These two strains were dumpy rollers (indicating a conversion event along with a deletion event in the *dpy-10* locus). We mated these two strains to CX12311 males to separate the *nurf-1* deletions from the *dpy-10* mutations.

### Dauer assays

Dauer assays were performed as described previously [8].

### Lifespan analysis

Lifespan assays were performed with standard method at 25°C using NGM plates containing 25 μM FUdR seeded with OP50. The animals were synchronized using alkaline-bleach to isolate embyros and raised on NGM plates at 20°C until they reached the young adult stage when they were transferred to FUdR plates at 25°C. The animal survival number was scored every two days. Animals were scored as dead when they no longer responded to gentle touch with a platinum wire. The date when the animals were placed on FUdR plate were defined at t = 0. The survival statistical analysis was performed in JMP12 software by using the log-rank method in Kaplan-Meier survival.

### Growth analysis

Animals were synchronized by allowing adults to lay eggs on an NGM plate seeded with OP50 bacteria for two hours and raised at 20°C. Three plates were created for each strain. At 48 and 72 hours, animals were recorded for one minute using a Videomach camera. Previously described tracking software was used to measure the area of each animal [33]. The average size of animals from each plate was normalized to the cumulative average size of the three N2 plates.

### HS-YE-HLE stock media preparation

The axenic liquid HS-YE-HLE stock media was prepared by mixing one volume of Heated Liver Extract (HLE) to nine volumes of HySoy-Yeast Extract (HS-YE). The preparation method is modified from the following paper [7]. To make the HLE component, calf liver (purchased from Corrina’s Corner) was cut to 1 inch squares and left in 4°C cold room overnight for 24 hours. An equal amount of distilled water was added to the liver, which was then further broken down using a blender. Large particles were filtered out of the homogenate with Miracloth. The purified homogenate was then heated in a 60°C water bath until its temperature reached 52°C and heated for 6 minutes. At that point the homogenate was centrifuge and the supernatant further filtered with a 0.2 micron filter (Nalgene). To prepare the HS-YE component, 40 g HySoy peptone (Sigma P6463) and 10 g yeast extract (Alfa Aesar H26769) was added to 1 L of water and autoclaved. The final HS-YE-HLE stock media also contained three antibiotics (Penicillin G 100 U/mL; Streptomycin 100 ug/mL and Amphotericin B 0.25 ug/mLand was filtered with a 0.2 micron filter (Nalgene).

### Competition assay

Competition assays between CX12311 and ARL_*nurf-1*_ (PTM88) were performed on NGM plates and liquid HS-YE-HLE stock media. A SNV located on chromosome I at position 11583395 (WS220) (CX12311: T, ARL_*nurf-1*_: C) was used to genotype and quantify the number of each strain in the competition experiments.

In the competition assay on NGM plates seeded with OP50 bacteria, nine experimental replicates were performed. Each replicate included five independent populations. At the beginning of each assay, the animals were synchronized with alkaline-bleach and raised at 20°C until they developed to the L4 stage. Seven L4 animals of each strain were placed on forty-five 6 cm NGM plates and kept at 20°C for one week. At this point, populations from each plate were transferred to new NGM plates by cutting a 0.5 cm x 0.5 cm square of agar (containing ~100 worms) from the starved plate. Populations were continuously cultured in this way for five weeks. During the six weeks of culturing, genomic DNA was isolated from the 2^nd^, 4^th^ and 6^th^ week time points. The populations from five plates in each group were combined into a single Eppendorf tube and genomic DNA was isolated by using a Qiagen Gentra Puregene Kit (cat. nos. 158667) following the supplementary protocol for nematodes and purified using Zymo Quick-DNA universal kit (cat. nos. D4068).

In the competition assay in liquid HS-YE-HLE stock media, six experimental replicates were performed. Each replicate was cultured in a 10 mL HS-YE-HLE stock media in a cell culture flask. At the beginning of this assay, the worms were synchronized by alkaline-bleach. When the animals were hatched, 100 L1 animals from each line were transferred into 10 mL of HS-YE-HLE media and cultured at 20°C in vertical shaker. After two weeks, 1 mL of depleted culture (containing ~2000 animals) were transferred to a new cell culture flask container. Then, the populations were continuously cultured for another four weeks. The populations were transferred every two weeks and the populations’ genomic DNA was isolated at the 2nd, 3^rd^, 4^th^, 5^th^ and 6^th^ week time point by the same method described above.

The proportion of ARL_*nurf-1*_ population size in competition assay was measured using Taqman analysis in Biorad QX200 digital PCR system to quantify the chromosome I 11583395 SNP frequency. Taqman probes were designed using standard software from Applied Biosystems. Genomic DNA from each time point was digested with SacI enzyme and purified with Zymo DNA Clean & Concentrator Kit (cas. nos. D4004). The concentration of fragmented genomic DNA was adjusted to 2 ng/uL by Qubit assay (cas. nos. Q32851). Digital PCR was performed followed the standard method provided by Biorad with the absolute quantification method. The proportion of ARL *nurf-1* allele was calculated and the linear model and statistical analysis were carried out in R programming language.

### High-throughput fecundity and growth rate assays

CX12311xLSJ2 RILs were assayed using a COPAS BIOSORT as described previously [50]. Abamectin (5 ng/mL) and albendazole (12.5 μM) were dissolved in DMSO, and arsenic trioxide (1 mM) and zinc chloride (350 μM) were dissolved in water. These treatments were paired with the respective solvent controls with a constant concentration of 1% volume-per-volume. The COPAS device outputs the number of objects, time of flight, extinction, and three different fluorescent parameters. These values were processed using a modified version of the COPASutils package [51] available on github.com/easysorter. The data were analyzed and plotted using R.

## Supporting Information

S1 Fig Analysis of sensitivities of various life history traits to four abiotics. **a.** QTL mapping of time of flight reaction norms (TOF) identifies a locus surrounding *nurf-1* for all four abiotics as measured by a COPAS BIOSORT. TOF is correlated with average length of the animals. **b.** QTL mapping of number of animal reaction norms (n) identifies a locus surrounding *nurf-1* for three of four abiotics as measured by a COPAS BIOSORT. n indicates the number of hatched progeny animals over the course of the experiment. **c.** Average number of progeny of RILs partitioned by their genotype at *nurf-1* (WBVar00601585) as measured by a COPAS BIOSORT in response to a variety of abiotic stressors.
(TIF)
