# Supplementary figures and images for: Selection on a Subunit of the NURF Chromatin Remodeler Modifies Life History Traits in a Domesticated Strain of Caenorhabditis elegans
Source: PLoS Genet. 2016 Jul 28;12(7):e1006219. doi: 10.1371/journal.pgen.1006219 (PMC4965130; doi:10.1371/journal.pgen.1006219)

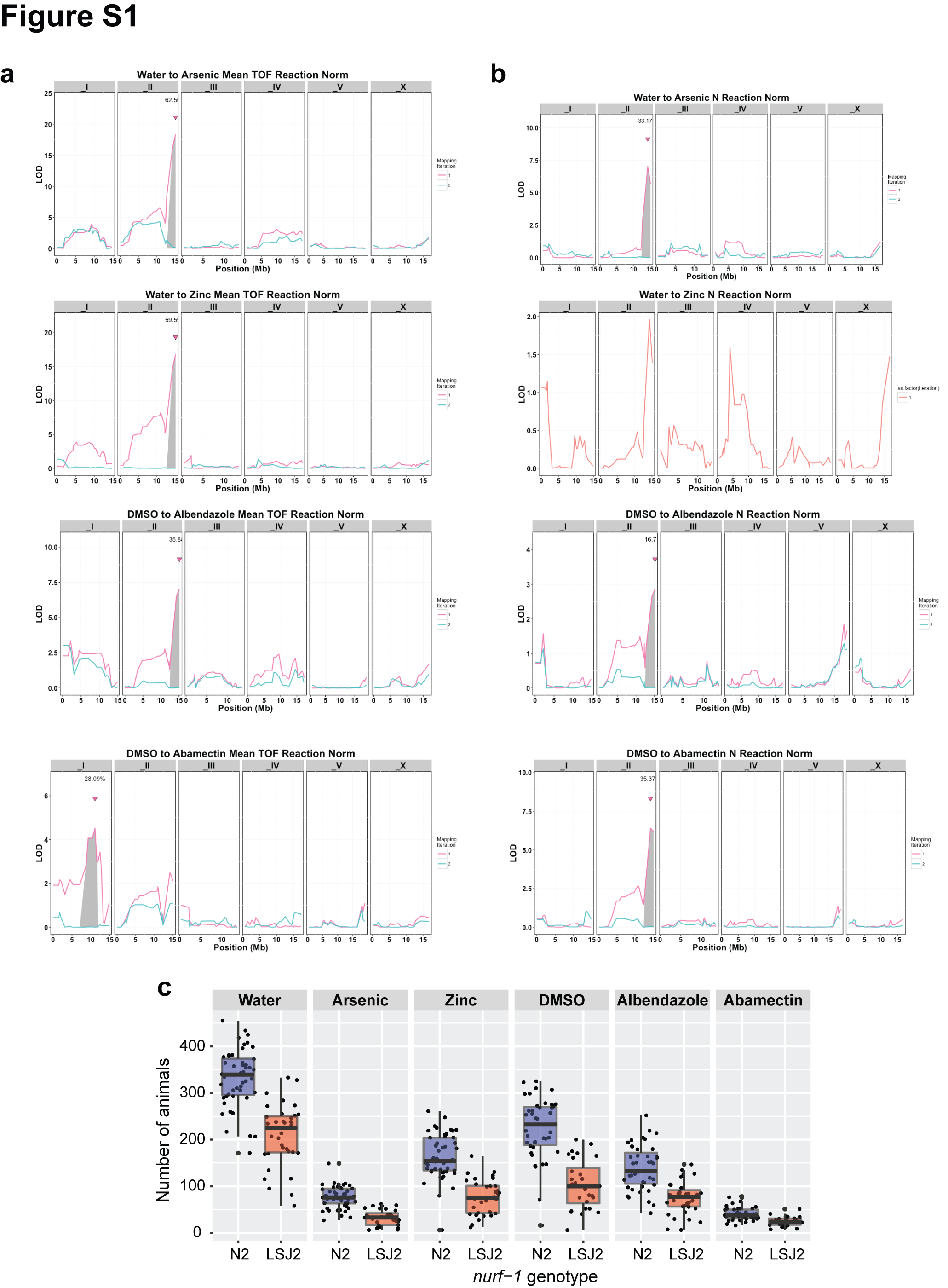

Supplement: S1 Fig — a. QTL mapping of time of flight reaction norms (TOF) identifies a locus surrounding nurf-1 for all four abiotics as measured by a COPAS BIOSORT. TOF is correlated with average length of the animals. b. QTL mapping of number of animal reaction norms (n) identifies a locus surrounding nurf-1 for three of four abiotics as measured by a COPAS BIOSORT. n indicates the number of hatched progeny animals over the course of the experiment. c. Average number of progeny of RILs partitioned by their genotype at nurf-1 (WBVar00601585) as measured by a COPAS BIOSORT in response to a variety of abiotic stressors. (TIF) [file pgen.1006219.s001.tif]
